# Perspectives of Black Patients on Racism Within Emergency Care

**DOI:** 10.1001/jamahealthforum.2024.0046

**Published:** 2024-03-08

**Authors:** Anish K. Agarwal, Rachel E. Gonzales, Charlotte Sagan, Sally Nijim, David A. Asch, Raina M. Merchant, Eugenia C. South

**Affiliations:** 1 Department of Emergency Medicine, Perelman School of Medicine, University of Pennsylvania, Philadelphia; 2 Leonard Davis Institute of Health Economics, University of Pennsylvania, Philadelphia; 3 Center for Health Care Transformation and Innovation, University of Pennsylvania, Philadelphia; 4 Perelman School of Medicine, University of Pennsylvania, Philadelphia; 5 Center for Health Justice, Penn Medicine, University of Pennsylvania, Philadelphia

## Abstract

**Importance:**

Numerous Black individuals experience racism persistently throughout their lives, with repercussions extending into health care settings. The perspectives of Black individuals regarding emergency department (ED) care, racism, and patient-centered approaches for dismantling structural racism remain less explored.

**Objective:**

To qualitatively explore the perspectives and experiences of Black patients related to race, racism, and health care following a recent ED visit.

**Design, Setting, and Participants:**

In this qualitative study, the audio from semistructured interviews of Black patients discharged from an academic urban ED between August 2021 to April 2022 were recorded, transcribed, and analyzed using thematic analysis.

**Main Outcomes and Measures:**

The main outcomes encompassed the main themes from the analysis of the interviews with Black patients regarding their perspectives on race, racism, and clinical care.

**Results:**

A total of 25 Black patients (20 [80%] female; mean [SD] age, 44.6 [12.9] years) discharged from the ED were interviewed. Three broad domains were identified: (1) racism in health care; (2) ED clinical care; and (3) recommendations for improvement. Within these domains, the first 2 were grouped into specific themes. Within the first domain, racism in health care, 7 themes were identified using thematic analysis: (1) a history of medical racism; (2) dismissiveness; (3) patient expectations on encountering racism; (4) medical mistrust; (5) health literacy; (6) postencounter outcomes, and (7) discrimination beyond but associated with race. Within the second theme, ED clinical care, 5 themes were identified using the same thematic analysis method: (1) discharge plan; (2) patient experience; (3) waiting room perceptions; (4) medication treatment; and (5) pain management. The third domain, recommendations for improvement, incorporated patient-generated suggestions for enhancing the Black patient experience.

**Conclusions and Relevance:**

In this qualitative study, the fabric of clinical care delivery in the ED was intricately woven with Black patients' experiences of racism. Patients expressed a pervasive sense of mistrust, skepticism, and dismissiveness at the system level. Instances of racism were consistently highlighted by patients from their entry to the ED to discharge. These perspectives illuminate the pervasive nature of racism in clinical care, providing valuable insights for exploring patient-centered approaches to foster antiracist cultures in the ED and throughout the broader medical landscape.

## Key Points

**Question:**

What are the perceptions, thoughts, and beliefs of Black patients on racism in emergency care?

**Findings:**

This qualitative study, including 25 interviews with Black patients discharged from the emergency department, described these patients’ perspectives about racism in health care, recent clinical experiences, and thoughts on system improvements. Black patients described a notable amount of medical mistrust, anticipation of racism in emergency care, and personal experiences with clinical instances of racism in emergency treatment.

**Meaning:**

These meaningful qualitative findings highlight clear and persistent racism in emergency care, suggesting opportunities to directly address racism in medicine and promote patient-centered approaches to improve equitable emergency care.

## Teaser

This qualitative study explores the perspectives and experiences of Black patients related to race, racism, and health care following a recent emergency department visit.

## Introduction

Racism in the US is an unrelenting force, ranging from racially targeted microaggressions to racism in foundational structures of education, housing, and employment.^1,2,3^ Racial discrimination pervades many aspects of society, including medicine and health care; more than 20% of people in the US reported experience with some form of discrimination related to medicine and health care. Racial discrimination is the most common form of bias reported by Black patients.^4^ When Black individuals enter health care spaces, racism can be explicitly or implicitly woven into the fabric of their care.^5,6^ Racism harms the health of Black people and is a root cause of racial health disparities.^7,8,9,10^

Evaluating and documenting the experience of Black patients within health systems is important; nevertheless, Black patients are rarely given the space to comment on their personal experiences and perspectives regarding race, health care, and how they intersect. Experiences with racism lead to heightened mistrust, poor communication between patients and their clinicians, and decreased engagement in preventive health services.^11,12,13,14^ Therefore, identifying and addressing racism in health care is a critically important aspect of providing quality health care, advancing equity, and dismantling structural racism.^3,5,10^

To eliminate racism within the walls of health systems, the system purveyors must understand patient perspectives to know where, when, and how these experiences are unfolding. Urban emergency departments (EDs) serve a critical role in serving historically marginalized Black communities, including individuals living in underresourced settings. These EDs also serve as the front door to a health system and provide a unique landscape to engage patients, investigate their experiences, and understand their perspectives.^15^ To begin to address this gap, we performed a qualitative analysis of Black patients’ perspectives on their ED encounters. Herein, we aim to explore the intersection of race, racism, and ED care by interviewing Black patients who were recently discharged from the ED.

## Methods

### Study Design and Participants

This qualitative study and thematic analysis incorporated semistructured interviews with Black patients discharged from the ED at an urban, academic, level 1 trauma center from August 10, 2021, to April 15, 2022. Interviews were designed to focus on patient perceptions, beliefs, and attitudes toward emergency care, health care, race, and experiences of racism in health care. This study was approved by the University of Pennsylvania’s institutional review board and exempted from written consent; however, verbal informed consent was obtained from patients. Adherence to the Consolidated Criteria for Reporting Qualitative Research (COREQ) reporting guideline was ensured.^14^

Study participants were recruited from the respondents of a parent study.^15^ In the aforementioned prospective cohort study, patients were recruited via text message within 24 hours of being treated and discharged from the ED to complete a mobile survey on experience. Patients who self-identified as Black in the mobile survey were invited to complete semistructured qualitative interviews. Interviews were conducted via telephone or on a virtual platform using audio only. Participants were compensated with a $30 gift card for their time.

### Study Procedure

Semistructured interviews with Black patients were conducted by 2 trained qualitative research team members (R.E.G. and C.S.). The study interviewers were not clinicians. The interviewers' demographics included 2 female researchers, of whom neither were Black, one identified as Hispanic, and one as multiracial. The interview guide was developed using existing frameworks related to racism and health by the study team, consisting of qualitative researchers and emergency physicians with expertise in equity (A.K.A., R.E.G., and E.C.S.; Supplement 1).^13,16^ The interview guide was developed by all members of the study team and pilot-tested by 2 authors (R.E.G. and C.S.) with other staff members, who self-identified as Black and were not clinicians. The guide included open-ended questions and subsequent probing questions, with a design that allowed participants to provide additional commentary.

The interview guide was based on how people may experience emergency care and medical racism, while also allowing for the nuances of experiencing individual racism and being aware of how an individual’s experience is shaped by structural forces. Of importance, the study procedure did not define racism in the script for participants, allowing for each individual to interpret the words racism, discrimination, and bias for themselves.

The interview guide was informed by 2 conceptual models related to racism. The first, created by Camara Jones, a pioneer in this space, determined the various levels of racism^17^: (1) institutional, which is a structural layer that deals with access to goods, services, and opportunities of society, including power patterned by race; (2) individual (personally mediated), which deals with prejudice (differential assumptions about another’s ability, motives, and intentions based on race) and discrimination (differential actions toward others based on race); and (3) internalized, which involves people from stigmatized races accepting negative messages about themselves. To parallel these concepts, the interview guide focused on perceptions of the health system and EDs, individual encounters within the ED, and viewpoints regarding racism and ED care. The second concept, based on the Everyday Discrimination Scale by David Williams, codifies how racism is experienced by individuals (eg, lack of respect, others acting as if you are dishonest).^16,18^ Using this framework, portions of the guide targeted communication between staff and the patient, feelings of respect toward the patient, and perceptions of care received throughout the visit. The audio of all interviews was recorded, transcribed, and deidentified using a professional transcription service and reviewed for accuracy.

### Thematic Analysis

A thematic analysis of interview responses was conducted using the constructivist paradigm, which is an approach aiming to understand a human phenomenon from the perspective of those individuals experiencing it.^19^ An initial codebook using a grounded theory approach for analyzing interview responses was developed, then revised iteratively throughout the coding process. Five interviews were initially coded twice, and the codebook was subsequently revised and updated to account for any discrepancies before additional coding was conducted. The revised codebook was applied to all transcripts, wherein the new code was a more appropriate categorization, known as a constant comparative coding approach. All interview transcripts were coded and analyzed by 2 trained qualitative research coders using NVivo qualitative analysis software, version 12 (Lumivero). The coders established strong interrater reliability accordance scores (κ > 0.8) and coded interviews independently. Interview length ranged from 20 minutes to 40 minutes with each participant, and the thematic content analysis of all 25 interview transcripts was reviewed by the research team. After review, the team determined that thematic saturation was reached. The thematic analysis was first performed for 20 interview transcripts, then again with all 25 interview transcripts to ensure no new codes or themes emerged.

## Results

A total of 25 Black patients (20 [80%] female; mean [SD] age, 44.6 [12.9] years) discharged from the ED were interviewed. Their characteristics are summarized in Table, including demographic data collected during the previous mobile survey.^15^ The semistructured interview guide is supplied in Supplement 1. Three broad domains were identified from participant responses: (1) racism in health care; (2) ED clinical care; and (3) recommendations for improvement. Within these domains, qualitative analysis revealed specific themes. Although some portions of these themes may intersect and overlap, they were described by participants as distinct areas. Within the first domain, racism in health care, 7 themes were identified using directed content analysis: (1) a history of medical racism; (2) dismissiveness; (3) patient expectations on encountering racism; (4) medical mistrust; (5) health literacy; (6) postencounter outcomes; and (7) discrimination beyond but associated with race. Within the second domain, ED clinical care, 5 themes were identified using the same method: (1) discharge plan; (2) patient experience; (3) waiting room perceptions; (4) medication treatment; and (5) pain management. Finally, a separate patient-generated domain, recommendations for improvement, was also identified. The domains, which are organized into themes with illustrative quotes, are presented in the Box.

**Table.** Participant Characteristics

Characteristic	No. (%)	
	Qualitative sample	Mobile survey sample^15^
Total No. of participants	25	462
Age, mean (SD), y	44.6 (12.9)	42.9 (17.3)
Sex		
Female	20 (80.0)	310 (67.1)
Male	5 (20.0)	152 (32.9)
Race		
Asian	0	16 (3.5)
Black	25 (100)	277 (60.0)
Multiracial	0	7 (1.5)
White	0	143 (31.0)
Not listed	0	12 (2.6)
Unknown	0	7 (1.5)
Education		
Some high school	1 (4.0)	NA
High school	3 (12.0)	NA
Some college	4 (16.0)	NA
College	4 (16.0)	NA
Some graduate school	1 (4.0)	NA
Not reported	11 (44.0)	NA

Abbreviation: NA, not analyzed.

Box. **Domains, Themes, and Illustrative Quotes**

**Domain 1: Racism in Health Care**

**A History of Medical Racism**

- “We’re super sensitive? Just take yourself out of your privilege and understand [what] our ancestors [have] been through and what we [are] still going through right now…Gentrification, cops shooting us, hospitals killing mothers, and taking our kids away, all types of stuff like that is going on right now in 2022. And then [to] say we’re sensitive, no, because we have to constantly be on guard from everything around us and not just these people, from our own people…” (Participant 12)

**Dismissiveness**

- “…I feel he was just passing me off. I don’t know if it’s he was just passing off what I said because he didn’t think that my pain was serious…Most times Black women aren’t taken seriously…but it is just like they say that most times we aren’t to be believed with our pain.” (Participant 16)

**Patient Expectations on Encountering Racism**

- “I know how that process goes, but it was because it’s like majority Black, it was…I could tell. And because I wasn’t dressed a certain way. How you look, you’re automatically treated a different way. You think I don’t know about my own health. You dismiss the answers in all, versus how someone else is a different race, dressed differently may look.” (Participant 15)
- “He doesn’t know anything about things that I’ve experienced…what I do for a living…places I’ve traveled in the world, or any of that. People have a prejudgment of me. But, like I said that day, I had just had surgery, so I hadn’t shaved. I’m looking scraggly. I just threw on some sweatpants and tried to get as comfortable as possible with the injury that I had. So, that might have played a part in his prejudgment of me as well. I can’t say it was solely race, but the thought crossed my mind more than once.” (Participant 11)

**Medical Mistrust**

- “I’ll just put it like this: seeking health care for a Black person is a roll of a dice all the time. [There will never] be a moment at this time in [the US] that a person of color [is] like, ‘Oh, I’ll just go to the doctor.’ No, it’ll be…‘Okay, what doctor am I going to? Is it bad enough that they’ll treat my condition [seriously], or should I wait until it’s bad enough that they have to?’ That’s a terrible way to live, and that’s how we live. We show up in the emergency room because everything else gets dismissed…so it gets bad enough that [there’s] no other choice until we have to get treated…it’s terrible.” (Participant 1)
- “Well, if you all know this, why am I not getting medicine for some type of relief, at least while I’m in the hospital? And…when I called the surgeon when I got home, he said, ‘Well, we try to stay away from that because we don’t want people to get addicted [to] opiates.’ I said, ‘Addicted? First of all, please check my record. I don’t even take medicine. I’m a breast cancer survivor. Don’t you know [that] I could be on medication?’…I’m not an advocate of swallowing pills, but were there instances in your life when you [were] enduring pain? And to talk about addiction? I’m the wrong person to talk about addiction. There is nothing in my profile that says anything about [addiction].” (Participant 18)

**Health Literacy**

- “So, if you think that I don’t have enough sense to tell somebody what I can and what they cannot use for me, you’re sadly mistaken. I’m very clear and very competent in explaining and expressing how I feel and what I can and cannot do. So, to automatically assume I don’t know anything about my health is crazy.” (Participant 18)
- “I think that they thought I didn’t know what I was talking about. Like I said, they went to [medical] school, and I didn’t…But I do read and…ask questions about the situation…I have learned a lot of things and read up on a lot of medication. Like I said, I’m still not a doctor, but I got common sense.” (Participant 3)

**Postencounter Outcomes**

- “If I had been treated like everybody else, I would’ve walked out of there with a sling and medication and a note for at least 3 days off. That’s standard procedure. I went in after midnight. That 1 day is the only day she wrote on that paper, knowing she gave me medication that was going to make me groggy, knowing I was going to be in pain for 3 days, and knowing I’m a health care provider…1 day off, no sling…and some medication. That, to me, is poor care.” (Participant 21)

**Discrimination Beyond but Associated With Race**

- “Yeah, from comparison I was saying. I also know, there was an assumption, but I think that also comes with race in the way in which presented. So I know how that process goes, but it was because it’s like majority Black, it was…I could tell. And because I wasn’t dressed, I think the race was in addition to how I dressed. How you look, you’re automatically treated a different way. You think I don’t know anything. You dismiss the answers in all, versus how someone else is a different race, dressed differently may look.” (Participant 15)
- “…It can’t always just be racism. It’s classism too. I see White people are dealing with it too…A lot of people…It’s not necessarily race anymore these days. It’s classism. And I’ve seen a man who was on his knees, and it is racism too, but I’m just saying, I don’t want to take that light out…We have people that have mental health issues, and they do things for attention. And the nurses, I’m pretty sure are trained to know these things. I don’t know. Because I kind of picked up on that too with him. But still, again, there are proper ways to address these types of people regardless, because you got all these people around you that [are] sick and in need [of] somebody soft to make sure that they’re OK. And the guy was on the floor, and she demanded, ‘Sir, you cannot be on the floor like that.’” (Participant 10)

**Domain 2: Emergency Department Clinical Care**

**Discharge Plan**

- “I didn’t really feel comfortable leaving…I was there waiting for a long time in my room…I’ve been having back pain for a while, and I hadn’t been seen by a doctor for a while…I had to go get [a] CT [computed tomography] scan, and I had to wait for a while for [the] CT scan results…They were like, ‘Oh, like everything’s fine. You can go.’” (Participant 22)

**Patient Experience**

- “I just think that had I been a different type of person, I’ll put it that way, that I don’t know if it was that I did home care or the color of my skin or what, that at least I would’ve been treated differently and better. She told me, she said, ‘You’ll probably feel pain for a few days. It’ll probably get worse, and then it’ll start getting better.’ It’s kind of like, we feel as though we aren’t being heard…We know what’s wrong with our bodies, but we don’t feel as though doctors and nurses take us seriously. I feel like they know and try to tell us…but they don’t take our pain seriously.” (Participant 14)

**Waiting Room Perceptions**

- “And so here…they’ll call me and they’re like, ‘Mom, I’ve gone in [at] 7, 8 o’clock in the evening, and now it’s 4 o’clock in the morning…I’m sitting there, and I’m watching along with [the] people of color, all the White people [had] gone.’” (Participant 7)
- “And it was only 3 people in the waiting room, 2 were taken, and I was sitting there. I [texted] my husband at work. I called both my sons and let them know that I was leaving. They know my MO [modus operandi]. I don’t tolerate disrespect. It doesn’t matter who it is, White, Black, purple, green. And I just thought a triage physician stating inaccurate information gave me a huge red light.” (Participant 19)

**Medical Treatment**

- “Oh no, [there] was one nurse, which I was thoroughly disgusted with…She came in, and she brought me an ibuprofen and muscle relaxers. And she couldn’t even tell me what the name of the muscle relaxers were. I don’t know if she just didn’t know because someone said, ‘Hey, this is the medicine for the woman in [room] 6.’ Or she was just looking at me like I should know. It was very weird and very bizarre. I looked, and I said, ‘Okay, I know that white pill right there is the ibuprofen. But what are these 2 peach-colored, small pills? What is that?’ Is this Percocet? Is it Vicodin? I didn’t know what those pills look like. I don’t take them. So I’m wondering what these medicines are while I’m in pain and trying to figure out what she’s giving me. And she was like, ‘It’s just your medicine. Take it.’ It was very cold, distant, and callous.” (Participant 17)

**Pain Management**

- “But I know what it is. I know what I felt. And that’s what it was from her. But otherwise, like I said, I give my visit an 8 out of 10 from those other nurses who came up and helped me inside of the emergency room area and then a guy who worked helping people find insurance at the front desk, an African American man. He was very nurturing, helpful. He helped me a lot, made me feel good. He had a nice personal story about his car accident that happened. And it was just nice. It’s nice when people are personable…when you share personable experiences with your patient.” (Participant 3)

**Domain 3: Recommendations and Suggestions for Improvement**

**Objective Support**

- “They need to put more people in the waiting room that look like us. They need to put more people that look like us behind the check-in counter and [those] taking care of us. It should not be all White people there.” (Participant 8)

**Health System**

- “You know what I would do? Like, if you said, I had a magic wand, I would just put cameras up. I don’t even think it’s a conscious thing. I would just put cameras up…in certain areas: triage, the discharge area, maybe the emergency room, wherever the treatment is, in general, just to show people the difference [between] when a person of color comes in and when a person [who] is not of color comes in…and make people watch themselves, and let them know everybody should be treated this way.” (Participant 9)

### Domain 1: Racism in Health Care

#### A History of Medical Racism

A central emergent theme repeated itself and overlapped with most of the other themes discussed in the interviews: patients described a history of medical racism. Medical racism was described by the participants as experiences of racism in the medical arena, as well as presumptions made by clinicians about Black patients, as well as any historical mention of medical racism, either their own experiences or those of others. Clear themes with distinct definitions emerged, yet participants commonly expressed that medical racism served as an underlying thread influencing their feelings and assumptions about medical care during an ED visit as Black patients.

#### Dismissiveness

Dismissiveness was the most expressed sentiment experienced by the interviewees. Patients felt that clinicians dismissed several concerns, including the severity of symptoms, pain levels, and treatment plan, especially about medication types and dosage or lack thereof. They also expressed frustration about clinicians not addressing their questions regarding their condition, its effects, and whether all necessary testing had been thoroughly conducted. Dismissiveness was also described as feeling like clinicians were not being attentive enough in their interactions with patients, such as participants feeling unheard or not being taken seriously, especially compared with other patients in the ED who belong to a different racial or ethnic group.

#### Patient Expectations on Encountering Racism

Patient expectations of experiencing racism during their ED care differed between participants. Some participants stated that they expected to encounter racism in the ED, due to either previous experience or general knowledge about the Black experience in medicine as a patient, while others reported they had no expectations before going to the ED. Participants who had family members working in medicine gained advanced knowledge of the day-to-day occurrences of medical racism and what to be aware of prior to their ED visit. Participants who discussed expectations of care anticipated dismissiveness, long wait times, and interaction with staff who were racially discordant. However, some participants with already familiar chronic illnesses found that they expected a treatment or form of screening that they did not receive.

#### Medical Mistrust

Participants discussed historical mistrust between the Black community and health care systems.^11,20^ Interviewees described perceptions of not feeling heard, listened to, or taken seriously by clinicians. Participants’ mistrust also became a factor in making a decision to seek care in the ED, as participants described feeling physically and psychologically unsafe within the ED as a Black individual. They further explained that the absence of Black clinicians and staff created an environment where they felt “othered.”

#### Health Literacy

Participants described attention to the perceived health literacy of Black patients as a consistent issue. Participants felt they were prejudged by clinicians for having low health literacy, no or low understanding of medical terms used by clinicians, and not heeding the advice of health care professionals. Many of the interviewees felt that medical staff made presumptions about their health, knowledge, and willingness to follow recommendations provided in the ED and that this affected their interactions and communication with the ED staff.

#### Postencounter Outcomes

Many reflected on how their ED experience had affected their view and faith in the health system, as well as the medical system in general. Some participants felt that their ED experience reaffirmed anticipated racism in health care, while others were cautious of receiving medical care unless it was truly emergent or life-threatening as they did not want to go through the experience of facing racism unless they had no other choice. Others discussed having transferred all of their medical information and care to this health system but that their experience led them to reevaluate that decision.

#### Discrimination Beyond but Associated With Race

Despite many participants feeling like their interactions in the ED were fueled by racism, other participants did not suspect race being a factor right away. These individuals instead attributed their experiences to different factors such as the perception of being treated poorly due to their financial or social class status, lower perceived education history, and their physical appearance (eg, clothing, weight, or hairstyle).

### Domain 2: ED Clinical Care

#### Discharge Plan

The discharge plan was a focal point for many participants. These individuals reported feeling that their discharge plan was not adequate for their diagnosis or did not connect with how they managed their own health. These specifically include the ability to follow up with a primary care physician or start new medications. Some interviewees also thought that their discharge plans substantiated their feelings of not being taken seriously and being dismissed by clinicians in the emergency department environment.

#### Patient Experience

The sentiment of this theme focused on the overall feelings of the participant either during or after their ED visit. Although sentiments tended to be overwhelmingly negative, participants also mentioned a few positive aspects. Among the negative sentiments, patients expressed feeling singled out due to racial identity, not fully heard by clinicians, and experiencing hostility from clinicians. Participants also discussed witnessing other Black patients who did not receive appropriate care or who were misinformed by clinicians. Several participants reported having such a negative experience in the ED that they no longer wanted to engage in the health care system further. Positive sentiments included brief ED interactions with triage staff and medical assistants, as well as a patient reflecting on care received from their routine care team at the University of Pennsylvania Health System.

#### Waiting Room Perceptions

Many of the interviewees mentioned long wait times in the ED as unfavorable experiences. A few discussed the waiting room being the first point of interaction within the ED, setting the tone for the rest of their experience. Patients reported that their wait times ranged from 2 hours to more than 8 hours. Many interviewees felt that their long wait times were directly associated with their race. Participants also reported that individuals who had arrived after them to the ED were seen first, with many having observed that those individuals were generally White patients. To this point, one participant reported observing all of the White patients being seen by the physician first, and the remaining patients left in the ED to be seen later were Black or belonged to another racial or ethnic minority group.

#### Medication Treatment

Medication was brought up in a few different ways in discussions with participants. First, some participants stated that medication was prescribed immediately to them without further care instructions or precautions taken. Second, some participants described being given acetaminophen or ibuprofen instead of stronger medications that they felt were needed based on their pain levels. Other participants discussed their concerns about lack of care and medication management from the medical staff.

#### Pain Management

Pain was a common concern among the interviewees. Participants who were experiencing severe pain reported waiting until their pain became unbearable or hard to ignore before going to the ED, and in some instances, the patients waited for pain to resolve on its own or treated pain with aspirin at home. Some participants also felt that their pain was downplayed by medical clinicians. When some participants discussed their pain, clinicians were hesitant to prescribe opioids or stronger medications, even when the patients believed that these treatments were needed to alleviate their pain. Some interviewees felt that clinicians made assumptions about patients’ pain levels, expressing their belief that Black and White patients were treated differently. Some participants mentioned that they read about the perception that Black people had higher pain tolerances and that this notion was previously taught in medical schools.

### Domain 3: Recommendations and Suggestions for Improvement

During the interviews, participants were asked what could have been done differently to improve their ED experience, as well as what the hospital could do to address racism and ensure equity among patients. Some participants brought up the idea of having an outside perspective or objective support (for example, cameras) to help validate their experiences in the ED related to their race. Others suggested that the health system needed to diversify the hospital staff at all levels, including triage specialists, nurses, and physicians.

## Discussion

In this qualitative study, which involved interviews with Black patients recently discharged from the emergency department, we explored the intersection of race, racism, and clinical care, revealing 3 notable findings. First, despite a recent shift towards prioritizing health equity and dismantling structural racism within hospitals over the past few years, experiences of racism have persisted within health care settings. The findings of this study illuminate Black patient perspectives from an urban ED, which may extend into other clinical environments. The study approach is crucial, as it identifies Black perspectives specific to emergency care and patient-inspired strategies for action. The participants in this study clearly described historic mistrust in medicine before even entering the ED^11,20^ and feeling dismissed and disappointed about the care they received while in the ED. These feelings and experiences extended beyond the ED walls, influencing patients’ reactions to encounters throughout the entire health system.

Second, perceptions of racism were woven throughout the fabric of clinical care delivery in the ED, which is often a primary point of care entry for historically marginalized communities, including patients of Black race.^15^ Race-based clinical care disparities continue to emerge in the literature related to pain management and are also being highlighted with the language used within the electronic health record.^21^ In this study, the participants described their experiences of being Black in the ED and perceptions of disparate care. These perceptions began in the waiting room (triage, time spent waiting),^22,23^ moved to the patient care area (hallway care), clinical interactions (dismissiveness), medical treatment (pain management), and continued during the discharge process (not being heard or fully evaluated). The linear pattern suggests that experiences of racism pervade many key clinical care points throughout ED encounters. Complex problems require deep investment and patient-centered solutions; thus, the continued push for antiracism efforts must be elevated as a priority for hospitals, clinics, and emergency departments.

Finally, Black patients in this urban setting were both skeptical of and invested in health systems. The setting of this study included an urban ED within a largely, historically Black and racially and economically segregated section of the city. Many participants described a long-standing history of health system mistrust and a sense of “not expecting much.” Participants also described a desire for the health system to improve equity in care delivery. Patients imagined scenarios to improve transparency to patients and accountability for health system leadership. One offered the notion of enhanced security cameras to document patient-to-clinician interactions. Additionally, patients noted the importance of equitable hiring across roles to increase diversity in the health care workforce and the clinicians who treat them.

To our knowledge, this study represents one of the first qualitative investigations specific to Black patients exploring their perceptions, beliefs, and experiences related to racism and acute care following a single ED encounter. This study identifies key patient perspectives related to perceptions of racism present in health systems for Black individuals in a historically segregated community, incorporating clinical pressure points specific to the ED. Moreover, EDs are uniquely positioned to serve marginalized, underrepresented communities facing discrimination, and they serve as an entry point for both emergency care and the larger health system. Often described as the front door, EDs may also function as critical ambassadors to elevate antiracist cultures in medicine.

### Limitations

First, the demographics of participants and the setting were exclusively focused on Black patients discharged from the ED within an urban, academic health system. Consequently, the results may not be generalizable to other populations or settings. In addition, participants were selected a priori based on Black race to focus the investigation on the racism encountered by Black patients. However, it is important to acknowledge that individuals of other races and ethnicities may have distinct experiences with racism in health care. Furthermore, because participants volunteered, there was a presence of sampling bias. Finally, the increased stress and strains within the ED due to the COVID-19 pandemic, along with hospital capacity challenges and violence in this setting at the time of this study, may have influenced responses. Nevertheless, the increased focus on improving equity and patient care in the ED made the work especially timely and important.

## Conclusions

In this qualitative study, Black patients reported varying experiences and perspectives on racism in an acute emergency care setting, but they commonly emphasized racism throughout the ED encounter and deep-seated medical mistrust. The historical presence of racism in medicine contributes to the prevailing mistrust of the health care system, persisting into the present. Substantial improvements in diversifying clinical staff, enhancing communication with patients, and ensuring equitable treatment are urgently needed to address perceptions of racism. Importantly, patients may experience both mistrust and a connection to health care institutions simultaneously. Thus, fostering more patient-centered collaboration will be essential to dismantle structural racism within medicine.

## Notes

Supplement 1. **eMethods.** Patient Interview Guide

Supplement 2.
Data Sharing Statement

## References

[1] Wong G, Derthick AO, David EJR, Saw A, Okazaki S. The what, the why, and the how: a review of racial microaggressions research in psychology. Race Soc Probl. 2014;6(2):181-200. doi:10.1007/s12552-013-9107-9 26913088 PMC4762607

[2] Sue DW, Capodilupo CM, Torino GC, . Racial microaggressions in everyday life: implications for clinical practice. Am Psychol. 2007;62(4):271-286. doi:10.1037/0003-066X.62.4.271 17516773

[3] Bailey ZD, Krieger N, Agénor M, Graves J, Linos N, Bassett MT. Structural racism and health inequities in the USA: evidence and interventions. Lancet. 2017;389(10077):1453-1463. doi:10.1016/S0140-6736(17)30569-X 28402827

[4] Nong P, Raj M, Creary M, Kardia SLR, Platt JE. Patient-reported experiences of discrimination in the US health care system. JAMA Netw Open. 2020;3(12):e2029650. doi:10.1001/jamanetworkopen.2020.29650 33320264 PMC7739133

[5] Hardeman RR, Medina EM, Kozhimannil KB. Structural racism and supporting Black lives—the role of health professionals. N Engl J Med. 2016;375(22):2113-2115. doi:10.1056/NEJMp1609535 27732126 PMC5588700

[6] US Institute of Medicine Committee on Understanding and Eliminating Racial and Ethnic Disparities in Health Care. Unequal treatment: confronting racial and ethnic disparities in health care. 2003. Accessed October 18, 2021. https://www.ncbi.nlm.nih.gov/books/NBK220358/

[7] Geronimus AT, Hicken M, Keene D, Bound J. “Weathering” and age patterns of allostatic load scores among Blacks and Whites in the United States. Am J Public Health. 2006;96(5):826-833. doi:10.2105/AJPH.2004.060749 16380565 PMC1470581

[8] DeSantis CE, Miller KD, Goding Sauer A, Jemal A, Siegel RL. Cancer statistics for African Americans, 2019. CA Cancer J Clin. 2019;69(3):211-233. doi:10.3322/caac.21555 30762872

[9] Gardener H, Sacco RL, Rundek T, Battistella V, Cheung YK, Elkind MSV. Race and ethnic disparities in stroke incidence in the Northern Manhattan Study. Stroke. 2020;51(4):1064-1069. doi:10.1161/STROKEAHA.119.028806 32078475 PMC7093213

[10] Khazanchi R, Evans CT, Marcelin JR. Racism, not race, drives inequity across the COVID-19 continuum. JAMA Netw Open. 2020;3(9):e2019933-e2019933. doi:10.1001/jamanetworkopen.2020.19933 32975568

[11] Armstrong K, Putt M, Halbert CH, . Prior experiences of racial discrimination and racial differences in health care system distrust. Med Care. 2013;51(2):144-150. doi:10.1097/MLR.0b013e31827310a1 23222499 PMC3552105

[12] Boulware LE, Cooper LA, Ratner LE, LaVeist TA, Powe NR. Race and trust in the health care system. Public Health Rep. 2003;(4):358-365. doi:10.1016/S0033-3549(04)50262-5 PMC1497554 12815085

[13] LaVeist TA, Isaac LA, Williams KP. Mistrust of health care organizations is associated with underutilization of health services. Health Serv Res. 2009;44(6):2093-2105. doi:10.1111/j.1475-6773.2009.01017.x 19732170 PMC2796316

[14] Trivedi AN, Ayanian JZ. Perceived discrimination and use of preventive health services. J Gen Intern Med. 2006;21(6):553-558. doi:10.1111/j.1525-1497.2006.00413.x 16808735 PMC1924636

[15] Agarwal AK, Sagan C, Gonzales R, . Assessing experiences of racism among Black and White patients in the emergency department. J Am Coll Emerg Physicians Open. 2022;3(6):e12870. doi:10.1002/emp2.12870 36570372 PMC9772489

[16] Williams DR, Yu Y, Jackson JS, Anderson NB. Racial differences in physical and mental health: socio-economic status, stress and discrimination. J Health Psychol. 1997;2(3):335-351. doi:10.1177/135910539700200305 22013026

[17] Jones CP. Levels of racism: a theoretic framework and a gardener’s tale. Am J Public Health. 2000;90(8):1212-1215. doi:10.2105/AJPH.90.8.1212 10936998 PMC1446334

[18] Krieger N, Smith K, Naishadham D, Hartman C, Barbeau EM. Experiences of discrimination: validity and reliability of a self-report measure for population health research on racism and health. Soc Sci Med. 2005;61(7):1576-1596. doi:10.1016/j.socscimed.2005.03.006 16005789

[19] Miles MB, Huberman AM, Saldaña J. Qualitative Data Analysis: A Methods Sourcebook. 3rd ed. SAGE Publications; 2014.

[20] Kennedy BR, Mathis CC, Woods AK. African Americans and their distrust of the health care system: healthcare for diverse populations. J Cult Divers. 2007;14(2):56-60. 19175244

[21] Sun M, Oliwa T, Peek ME, Tung EL. Negative patient descriptors: documenting racial bias in the electronic health record. Health Aff (Millwood). 2022;41(2):203-211. doi:10.1377/hlthaff.2021.01423 35044842 PMC8973827

[22] Pitts SR, Pines JM, Handrigan MT, Kellermann AL. National trends in emergency department occupancy, 2001 to 2008: effect of inpatient admissions versus emergency department practice intensity. Ann Emerg Med. 2012;60(6):679-686.e3. doi:10.1016/j.annemergmed.2012.05.014 22727201

[23] Pines JM, Russell Localio A, Hollander JE. Racial disparities in emergency department length of stay for admitted patients in the United States. Acad Emerg Med. 2009;16(5):403-410. doi:10.1111/j.1553-2712.2009.00381.x 19245372

