# Supplementary material for: Perspectives of Black Patients on Racism Within Emergency Care
Source: JAMA Health Forum. 2024 Mar 8;5(3):e240046. doi: 10.1001/jamahealthforum.2024.0046 (PMC10924244; doi:10.1001/jamahealthforum.2024.0046)
Supplement: Supplement 1. — eMethods. Patient Interview Guide [file jamahealthforum-e240046-s001.pdf]

Supplemental Online Content

Agarwal AK, Gonzales RE, Sagan C, et al. Perspectives of Black patients on racism within emergency care. *JAMA Health Forum*. 2024;5(3):e240046. doi:10.1001/jamahealthforum.2024.0046

eMethods. Patient Interview Guide

This supplemental material has been provided by the authors to give readers additional information about their work.

© 2024 Agarwal AK et al. *JAMA Health Forum*.

eMethods. Patient Interview Guide
Patient Interview Script, version 2

April 29, 2021

<u>INTRODUCTION</u>

Hello, my name is ______. I am from the University of Pennsylvania and am working with a team of doctors who want to know more about the experiences of patients who have gotten care our emergency department (also called the ED or the ER). In particular, our department has a goal to promote health equity, and so we want to hear from our Black patients.

We know from prior research that some patients experience racism and discrimination in hospitals and doctors offices across the country. Our goal is to use your feedback and the feedback of about 30 other Black patients who we are interviewing, to improve care in the ER for all patients and specifically to reduce experiences of racism. We want you to be completely honest.

Before we start our interview it's important to go over a few things.
1. This interview will take about 30-45 minutes.
2. This interview is completely confidential and will not be shared with your doctors. The answers you give here will not impact your current or future care at Penn Medicine. You are free to share as much or as little about your experiences as you would like, and we can skip a question or stop the interview at any point.
3. We will record and then transcribe the interview. All identifying information will be removed and we will destroy the recording. Your name and any identifying information will never be used in connection with the information from your survey or this interview.
4. Participation in this research is completely voluntary. You may opt out at any time if you wish.
5. You will receive a $30 gift card for your time.

<u>QUESTIONS</u>

<u>Part I (Perceptions of the ER)</u>

First we want to talk about your general impressions of the ER at Penn Medicine. Specifically whenever we say ER, we are talking about either at Presbyterian Medicine Center (or Presby at 38th and Powelton) or the Hospital of the University of Pennsylvania (or HUP at 34th and Spruce)

1. Think back to the first time you ever went to the ER at Penn Medicine as a patient or the family member of a patient. When was that? Do you remember why you were there? What was your experience like?

2. When you and your family or friends talk about the ER at Penn Medicine - what do you talk about?
    - What are the positive things you talk about?
    - What are the negative things you talk about?
        i. Have you or your family ever been concerned about racism or discrimination in the Penn ERs?
    - What do you expect your experience to be like if you or a family member have to go the ER at Penn?
    - How do you feel when you talk about the ER at Penn with your family or friends?

© 2024 Agarwal AK et al. *JAMA Health Forum*.

3. Tell us about your connections to West or Southwest Philly? How long have you lived here? Did you grow up here?
    > [if they grew up in W/SW] Do you remember hearing anything about the ER when you were growing up?

Part II Last visit to ER

Now, we want to talk about your visit to the [PMC/HUP] ER on [insert date]. Tell me about that day.

1. First, what was going on before you went in and why did you decide to go? What concerns, if any, did you have any about going?
    > Probes: Why do you think you had those concerns?

Now we would like to hear about care you received. We will talk about your overall experience and then specific aspects of your visit and interaction with the staff.

The first few things that often happens when a patient comes to the ER is they are checked in by registration and then they talk to a nurse and/or doctor briefly as part of the triage process. All of this happens at the front of the ER before a patient goes into a room.

2. What happened when you came to the ED? Tell me about the whole experience from the moment you walked in, to the moment you left?? Please describe in as much detail as you are comfortable.
    > What do you remember about these initial moments in the ER?
    > Was this a first time visit to this ED?
    > Can you describe each step of your journey and who you interacted with?
    > Were there specific people that you remember? Why?
    > Was there anything about these initial moments that did not go well for you?
    > Did you spend any time in the waiting room? What was that like for you?
    > Probe stages: check in/triage, waiting room, getting a room/hallway bed, in room, discharge, follow-up
3. Tell me about the communication you had with your doctors, nurses, and other staff in the ER during the visit.
    > Did you ever feel that your doctors or nurses were not listening to what you had to say about your health concerns?
    > Did you ever feel that your doctors or nurses acted as if they didn't think you were smart?
    > Do you think the manner with which your doctors or nurses communicated with you was affected because of your race? Tell us more about that . . . .

Clinical care

4. Please describe the clinical care you received. By clinical care I mean labs and tests and medications that you got to help figure out what was going on with your health and treat your symptoms. How was your experience getting your health issue looked after?

5. Do you think any aspects of your clinical care – what tests/medications you did or did not get – do you think it was related to your race?
    > If yes, why did you answer this way?
    > If no, why did you answer this way?

© 2024 Agarwal AK et al. *JAMA Health Forum*.

6. Did you notice any police or law enforcement in the ER the day you were there? What did you think about their presence? Does this make you more or less comfortable or safe in the ER?

Overall treatment & respect

7. Overall, do you think you were treated with respect as a patient in your visit?
   > Tell us more about why or why not.
   > What do you think the doctors or nurses thought about your and why you were in the ER that day?
   > Do you think any of them thought they were better than you? Or afraid of you?
   > Did any of the ER staff or care team act as if they thought you were dishonest?
8. What made you most comfortable during your ER visit? What made you most uncomfortable during your ED visit?
9. In your overall visit and experience, did you experience any racism, bias or discrimination? Tell us more about this.

Impact

10. How has your experience during your last visit impacted your view on healthcare services?
11. Will your experience during your last visit impact how you seek healthcare services again?
    > Probes:
    1. Taking medication
    2. Following care team's instructions
    3. Coming in for care in the future
    4. Perceptions of healthcare system overall

<u>Part III (Future Considerations)</u>

Now we are very interested to hear your suggestions for how care for patients can be improved, especially regarding reducing racism and discrimination in the ED.

12. What could have been differently during your ER visit to make it a better experience?
    > Proves: (Depending on other answers: initial registration, triage, waiting room, time in room, clinical care, discharge process, etc.)
    > What would you change about the way the ED provided care for patients?
13. What could the hospital do to address racism and ensure patients are treated fairly?

Thank you for participating in this interview.

Brief Demographic Collection:

- Age
- Sex
- Race
- Ethnicity
- Level of education
- Housing status
- Phone status
- Reason for ED visit

© 2024 Agarwal AK et al. *JAMA Health Forum*.
