# Supplementary material for: Perspectives of Black Patients on Racism Within Emergency Care
Source: JAMA Health Forum. 2024 Mar 8;5(3):e240046. doi: 10.1001/jamahealthforum.2024.0046 (PMC10924244; doi:10.1001/jamahealthforum.2024.0046)
Supplement: Supplement 2. — Data Sharing Statement [file jamahealthforum-e240046-s002.pdf]

Data Sharing Statement

Agarwal. Perspectives of Black Patients on Racism Within Emergency Care. *JAMA Health Forum*. Published March 08, 2024. doi:10.1001/jamahealthforum.2024.0046

Data
Data available: Yes
Data types: Deidentified participant data
How to access data: anish.agarwal@pennmedicine.upenn.edu
When available: With publication

Supporting Documents
Document types: None

Additional Information
Who can access the data: researchers whose proposed use of the data has been approved
Types of analyses: for a specified purpose
Mechanisms of data availability: after approval of a proposal
